# Supplementary material for: From sequence to enzyme mechanism using multi-label machine learning
Source: BMC Bioinformatics. 2014 May 19;15:150. doi: 10.1186/1471-2105-15-150 (PMC4229970; doi:10.1186/1471-2105-15-150)
Supplement: Additional file 2 — Java code of ml2db. Additional file ml2db_code.tar.gz contains the Java source code to run the multi-label machine learning experiments and save the results to database. The code’s Javadoc is included. [file 1471-2105-15-150-S2.zip › additional file 2/ml2db/ml2db/doc/main/ml2db/weka/package-summary.html]

main.ml2db.weka


JavaScript is disabled on your browser.


- Overview
- Package
- Class
- Use
- Tree
- Deprecated
- Index
- Help

- Prev Package
- Next Package

- Frames
- No Frames

- All Classes

# Package main.ml2db.weka

- Class Summary

  | Class | Description |
  | --- | --- |
  |  |  |
  | --- | --- |
  | Arff | Creates an ARFF file: checks file properties, loads data from database, fills and writes to file the ARFF attributes, classes and instances, generates auxiliary files if needed. |
  | ArffProps | A class containing the meta-data for a Weka ARFF file (or arff + xml files for Mulan ARFF) The data includes the path to an existing ARFF file, an optional data set name and description, and various statistics for the data (number of instances, class values, attributes) |
  | ArffPropsFromFile | Contains the properties for a Weka ARFF file to be generated from database queries. |
  | ArffRecordsTable | Class to store the name and column types for the database table which stores the ARFF records. |
  | AttributeFactory | The attribute factory produces binary and numeric Weka attributes. |
  | DataSetChecker | Checks that all data (or file paths) needed to build the ARFF file are available |
  | DataSetDbLoader | Loads data (instances, attribute values, class values) from database using the queries in the data Properties. |
  | DataSetGenerator | Generates and fills the Weka instances. |
  | DataSetManager | DataSet generates a collection of Weka instances (rows of the ARFF file), their attributes (column names) and their attribute values (cell values) The last attribute is the class attribute. |
  | DataSetWriter | Class |
  | IdentifiedSparseInstance | Class |
  | ImportArff | Class to import an existing Weka ARFF file to a record in the arff database table. |
  | InstancesFiller | Generates and fills individual instances for a weka data set. |
  | InstAttValueMap | Contains [instance identifier, attribute name, attribute value] tuples. |
  | JaccardDistance | This is an implementation of the Jaccard distance which calculates it on the fly. |
  | JaccardDistanceStored | This is an implementation of the Jaccard distance which pre-calculates and stores the numbers needed. |
  | JaccardDistanceTest |  |
  | JaccardEuclideanDistance | A Jaccard distance. |

- Overview
- Package
- Class
- Use
- Tree
- Deprecated
- Index
- Help

- Prev Package
- Next Package

- Frames
- No Frames

- All Classes
